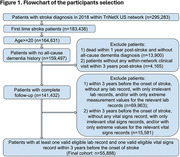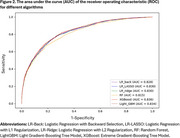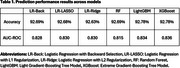# Comparing Machine Learning and Traditional Models to Predict One‐Year Post‐Stroke Dementia Risk

**DOI:** 10.1002/alz70860_106285

**Published:** 2025-12-23

**Authors:** Xueting Ding, Jiahui Dai, Liner Xiang, Yang Meng, Bruce Albala, Alissa Kurzman, Megan Castro, Desiree Gutierrez, Bernadette Boden‐Albala

**Affiliations:** ^1^ Joe C Wen School of Population & Public Health, Henry and Susan Samueli College of of Health Sciences, University of California, Irvine, Irvine, CA, USA; ^2^ Joe C. Wen School of Population & Public Health, Henry and Susan Samueli College of Health Sciences, University of California, Irvine, Irvine, CA, USA; ^3^ Donald Bren School of Information & Computer Sciences, University of California, Irvine, Irvine, CA, USA

## Abstract

**Background:**

Alzheimer's disease and related dementias (ADRD) are characterized by memory impairment, loss of independence, and increased mortality risk. Stroke is a well‐established risk factor, with approximately one in three stroke survivors developing ADRD. The risk is highest in the first year post‐stroke, when patients face triple the typical risk. Although this risk decreases after 12 months, it remains elevated for up to 20 years. However, limited research has explored machine learning algorithms with real‐world clinical data to predict post‐stroke dementia. Thus, this study aimed to assess various predictive modeling approaches to identify ADRD risk within one year after stroke using electronic health records (EHR).

**Method:**

We extracted EHR data from the TriNetX Network. Our study included adult patients (age ≥20 years) who experienced their first stroke in 2018. Figure 1 presents our detailed patient selection criteria. Our primary outcome was ADRD occurring within one year after stroke. We incorporated demographic characteristics, vital and laboratory measurements, comorbid conditions, and medication history in our models. We compared six prediction models: traditional logistic regression with backward selection (LR‐Back), regularized regression (LR‐LASSO and LR‐Ridge), Random Forest (RF), Light Gradient‐Boosting Tree Model (LightGBM), and Extreme Gradient‐Boosting Tree Model (XGBoost). We evaluated model performance using classification accuracy and area under the receiver operating characteristic curve (AUC‐ROC).

**Result:**

Among 55,888 stroke patients in our final cohort, 8% developed ADRD in the year following their stroke. The cohort included 48% females and had a predominant elderly population (54% aged 65+), followed by middle‐aged adults (37% aged 45‐64) and younger adults (9% aged 20‐44). 64% were Non‐Hispanic Whites. Among ADRD cases, half were female and 81% were 65+, with higher systolic blood pressure, lower BMI, and greater prevalence of comorbidities and medication use. All models demonstrated strong predictive performance, with accuracy exceeding 92% (Table 1, Figure 2). The XGBoost model achieved the highest performance metrics (accuracy: 92.78%, AUC: 0.836), closely followed by LightGBM (92.78%, 0.834).

**Conclusion:**

Our findings demonstrate that both machine learning and traditional statistical approaches may effectively predict one‐year post‐stroke dementia risk using EHR data, which could help identify high‐risk patients for early monitoring.